# Does diabetes affect breast cancer survival?

**DOI:** 10.1002/cnr2.2040

**Published:** 2024-03-20

**Authors:** Ross Lawrenson, Chunhuan Lao, James Stanley, Andrea Teng, Marion Kuper‐Hommel, Ian Campbell, Jeremy Krebs, Dianne Sika‐Paotonu, Jonathan Koea, Ineke Meredith, Jason Gurney

**Affiliations:** ^1^ Medical Research Centre The University of Waikato Hamilton New Zealand; ^2^ Commissioning Te Whatu Ora Waikato Hamilton New Zealand; ^3^ Department of Public Health University of Otago Wellington New Zealand; ^4^ Medical Oncology Waikato Hospital Hamilton New Zealand; ^5^ Department of Surgery, Faculty of Health Sciences The University of Auckland Auckland New Zealand; ^6^ Department of Medicine University of Otago Wellington New Zealand; ^7^ Dean's Department UOW & Division of Health Sciences University of Otago New Zealand; ^8^ General Surgery Waitakere Hospital Auckland New Zealand; ^9^ Medical Surgery The University of Auckland Auckland New Zealand; ^10^ General Surgery Wakefield Hospital Wellington New Zealand

**Keywords:** breast cancer, cause of death, diabetes, mortality, survival

## Abstract

**Objectives:**

The objective of this study is to investigate the influence of diabetes on breast cancer‐specific survival among women with breast cancer in Aotearoa/New Zealand.

**Methods:**

This study included women diagnosed with invasive breast cancer between 2005 and 2020, with their information documented in the Te Rēhita Mate Ūtaetae—Breast Cancer Foundation National Register. Breast cancer survival curves for women with diabetes and those without diabetes were generated using the Kaplan–Meier method. The hazard ratio (HR) of breast cancer‐specific mortality for women with diabetes compared to women without diabetes was estimated using the Cox proportional hazards model.

**Results:**

For women with diabetes, the 5‐year and 10‐year of cancer‐specific survival were 87% (95% CI: 85%–88%) and 79% (95% CI: 76%–81%) compared to 89% (95% CI: 89%–90%) and 84% (95% CI: 83%–85%) for women without diabetes. The HR of cancer‐specific mortality for patients with diabetes compared to those without diabetes was 0.99 (95% CI: 0.89–1.11) after adjustment for patient demographics, tumor characteristics, and treatments. Age at cancer diagnosis and cancer stage had the biggest impact on the survival difference between the two groups. When stratified by cancer stage, the cancer‐specific mortality between the two groups was similar.

**Conclusions:**

While differences in survival have been identified for women with diabetes when compared to women without diabetes, these are attributable to age and the finding that women with diabetes tend to present with more advanced disease at diagnosis. We did not find any difference in survival between the two groups due to differences in treatment.

## 1 INTRODUCTION

Breast cancer is the most prevalent and widely diagnosed malignancy for women in Aotearoa/New Zealand.^1^ Approximately 5% of breast cancer patients have metastatic disease at diagnosis (de novo metastatic breast cancer),^2^ where the prognosis for these patients is poor, with a 5‐year breast cancer‐specific survival of only 26% for de novo metastatic breast cancer.^3^ In contrast, the outcome for women diagnosed with stages I–III breast cancer is favorable, with a 5‐year breast cancer‐specific survival rate exceeding 90% for these women.^4^ Advanced stage at diagnosis (stage III and IV) has been found to make the greatest contribution to the difference in breast cancer‐specific survival by ethnicity,^5^, ^6^ suggesting/making reducing the proportion of patients diagnosed with advanced breast cancer the key to improving ethnic disparities in cancer survival.

Diabetes is a multisystem disorder that is associated with a greater risk of getting breast cancer.^7^, ^8^, ^9^, ^10^ In Aotearoa/New Zealand, the rate of breast cancer is approximately 15% higher among those with diabetes than people without diabetes.^10^ Patients with diabetes often have worse tumor characteristics at cancer diagnosis, and poorer outcomes when compared to patients without diabetes.^11^, ^12^ For example, women with diabetes have been found to have a higher risk of having metastatic disease at breast cancer diagnosis when compared to those without diabetes (odds ratio [OR]: 1.17, 95% confidence interval [CI]: 1.00–1.38).^11^ Diabetes impacts on various aspects of breast cancer treatment decisions and timelines for women across different cancer stages. For women with stages I–III breast cancer, the presence of diabetes diminishes the likelihood of undergoing breast reconstruction following mastectomy (OR: 0.54 for stage I cancer, 0.50 for stage II, and 0.48 for stage III cancer). Additionally, diabetes is associated with prolonged surgical delays, as indicated by an OR of 1.16 (95% CI: 1.05–1.27).^2^ We also found that women with diabetes were less likely to have chemotherapy, but that women with diabetes were more likely to receive endocrine therapy for stage III disease and had better adherence to endocrine therapy.^13^, ^14^

The impact of diabetes on survival in people with breast cancer has not been examined in Aotearoa/New Zealand, and findings from international studies are controversial.^15^, ^16^, ^17^, ^18^, ^19^, ^20^ Some studies have shown that diabetes is associated with an elevated risk of all‐cause mortality,^15^, ^16^ while contrasting studies have reported no discernible link between diabetes and either all‐cause mortality or breast cancer‐specific survival.^17^, ^18^, ^19^, ^20^ Evidence suggests that diabetes increases the risk of breast cancer recurrence.^20^ The objective of this study is to examine the impact of diabetes on breast cancer‐specific survival and metastatic relapse in Aotearoa/New Zealand, for people with breast cancer.

## 2 METHODS

This research investigated breast cancer outcomes on a national scale for women who received a diagnosis of invasive breast cancer between 2005 and 2020 recorded in Te Rēhita Mate Ūtaetae—the Breast Cancer Foundation National Register (NBCR). The NBCR captures diagnostic and treatment details for 99.5% of breast cancer cases in Aotearoa/New Zealand, encompassing both public and private healthcare facilities. The NBCR provides comprehensive clinical data related to breast cancer. It captures a range of patient demographics, including age at diagnosis, menopausal status (pre‐menopause, peri‐menopause, and post‐menopause) and ethnicity. Tumor information, such as diagnosis date, mode of detection (screen detected or symptomatic), TNM cancer stage (I, II, III, and IV), grade (1, 2, and 3), and biomarkers (estrogen receptor [ER], progesterone receptor [PR], and human epidermal growth factor receptor 2 [HER2]), are comprehensively documented. HER2+ is defined as HER2:3+ and/or HER2 amplified by FISH.^23^ Detailed information on treatment modalities, including surgery, radiotherapy, chemotherapy, endocrine therapy, and HER2‐targeted therapies, is recorded. Breast cancer outcomes, specifically cancer recurrence and mortality, are also part of the recorded data.

The NBCR data are linked to the virtual diabetes register (VDR) to ascertain the diabetes status (whether the individual has diabetes or not) at the time of breast cancer diagnosis. The VDR, a national register, provides an estimate of diagnosed diabetes prevalence in Aotearoa/New Zealand. It does not differentiate between type 1 and type 2 diabetes, and the date of diabetes diagnosis is not included in the records. Patients are categorized as having diabetes if they have ever been documented in the VDR before their cancer diagnosis. The National Minimum Dataset (NMDS) was used for the identification of comorbidities, as documented in hospital admission records. The NMDS serves as a comprehensive repository of public and private hospital discharge data, encompassing coded clinical information for both inpatients and day patients. Linkages are also established with the Mortality Collection and Death Certificates to ascertain both the date and underlying cause of death. Data linkages are facilitated through the National Health Index (NHI) number, a distinctive identifier assigned to individuals accessing healthcare services in Aotearoa/New Zealand.

Ethnicity is self‐identified in these datasets, and for the purpose of this study, we have classified it into four groups (Asian, European/Other, Māori, and Pacific) following the guidelines outlined in the Ethnicity Data Protocols.^21^ Socioeconomic status was determined using the area‐based New Zealand Index of Deprivation 2018 (NZDep 2018), analyzed in quintiles ranging from 1 (least deprived) to 5 (most deprived).^22^ Comorbid conditions were extracted from the NMDS for hospitalizations within the 5 years leading up to and including the index hospitalization date. This information was used to calculate a C3 index score for each patient. The C3 index serves as a cancer‐specific measure of comorbidity, with scores categorized as “0” (⩽0), “1” (⩽1.00), “2” (⩽2.00), and “3” (>2.00).^23^ Notably, diabetes was excluded as a comorbidity from the C3 index score calculation. Breast cancer subtypes were categorized into five groups based on biomarker status.^24^

Luminal A: ER+, PR+, and HER2−
Luminal B HER2‐: ER or PR+ (but not both +), HER2−
Luminal B HER2+: ER+ and/or PR+, HER2+
HER2 non‐Luminal: ER−, PR−, HER2+
Triple negative: ER−, PR−, HER2−

The Mortality Collection classifies and codes the underlying cause of death for all deaths registered in Aotearoa/New Zealand, and the death certificates stores uncoded cause of death data and does not record the underlying cause of death. We examined the death certificates to determine the underlying cause of death for these patients, defined by the World Health Organization (WHO) as “(a) the disease or injury which initiated the train of events leading directly to death, or (b) the circumstances of the accident or violence which produced the fatal injury.”^25^ We compared the underlying cause of death between women with diabetes and women without diabetes. The underlying cause of death was categorized into nine groups: (1) breast cancer; (2) cardiovascular and cerebrovascular disease (CVD); (3) other cancers; (4) respiratory disease; (5) diabetes; (6) infection; (7) renal failure; (8) others; and (9) unknown cause.

For survival analysis, patients were censored at the date of death, or on December 31, 2020 (the last follow‐up date for this study). For death from breast cancer, date of death was the event time, while for death from other causes, people were censored at date of death. Kaplan–Meier methods were employed to generate breast cancer survival curves, reporting 5‐year and 10‐year survival rates with 95% CI, for both women with and without diabetes. Cox proportional hazards models were utilized to estimate the unadjusted hazard ratio (HR) of breast cancer‐specific mortality for women with diabetes in comparison to those without diabetes. The analysis incorporated various factors, including age, cancer stage, and other patient characteristics and treatments. Adjusted factors were introduced one by one in the models. These analyses were conducted for the entire patient cohort and were subsequently stratified based on cancer stage and ethnicity, providing a comprehensive examination of the impact of diabetes on breast cancer‐specific mortality across different subgroups.

For the metastatic relapse analysis, among patients with stages I–III breast cancers at the index diagnosis, any distant metastatic recurrence was defined as metastatic relapse and the date of first distant metastatic recurrence was considered as the outcome date. Patients who did not have metastatic relapse during the follow‐up time were censored at the date of death, or on December 31, 2020. The HR of metastatic relapse for patients diagnosed with stages I–III breast cancer was calculated both before and after stratification based on cancer stage.

All data analyses were conducted using R 4.0 (R Institute, Vienna, Austria) and the Survival package. The research protocol received ethical approval from the University of Waikato Human Research Ethics Committee, with the reference number HREC(Health)2021#89, ensuring adherence to ethical standards and guidelines in the execution of the study.

## 3 RESULTS

### 3.1 Underlying cause of death

Throughout the study period of 2005–2020, a total of 26 968 women had a diagnosis of breast cancer, and 3137 women, constituting 11.6% of the patient population, were identified as having diabetes at the time of their cancer diagnosis. Patient demographics and tumor characteristics are display in Table A1. By the end of 2020, there were 843 deaths in the diabetes group including 44% of breast cancer deaths and 4119 deaths in the non‐diabetes group including 60% breast cancer deaths (Table 1). Among other causes of death, CVD is the main underlying cause of death for both women with diabetes (22%) and women without diabetes (13%), followed by other cancers (10% with diabetes and 11% without diabetes) and respiratory disease (5% with diabetes and 5% without diabetes). Metastatic disease only accounted for 5% among women with breast cancer and diabetes, and 7.4% among women with breast cancer and without diabetes. However, 38.2% of the breast cancer deaths in women with diabetes were from patients with metastatic disease at primary diagnosis, and this figure was 30.1% for women without diabetes.

**TABLE 1:** Underlying cause of death between women with diabetes and women without.

Cause of death	Diabetes		No diabetes		Total	
	*n*	Prop	*n*	Prop	*n*	Prop
Breast cancer	372	(44%)	2467	(60%)	2839	(57%)
CVD	187	(22%)	554	(13%)	741	(15%)
Other cancers	84	(10%)	443	(11%)	527	(11%)
Respiratory disease	40	(5%)	213	(5%)	253	(5%)
Diabetes	41	(5%)	0		41	(1%)
Infection	9	(1%)	24	(1%)	33	(1%)
Renal failure	6	(1%)	7	(0.2%)	13	(0.3%)
Others	92	(11%)	366	(9%)	458	(9%)
Unknown	12	(1%)	45	(1%)	57	(1%)
All deaths	843		4119		4962	

### 3.2 Survival rate

**FIGURE 1:**
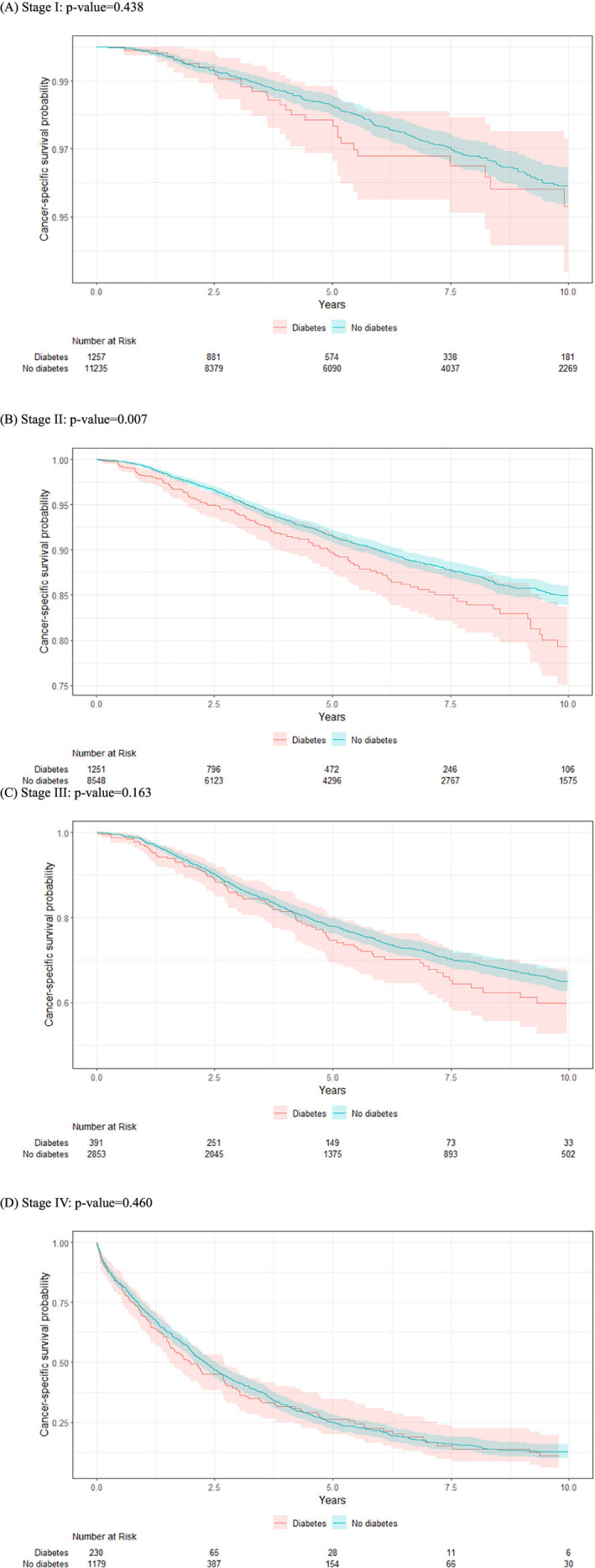
Breast cancer‐specific survival by diabetes status: (A) Stage I; (B) Stage II; (C) Stage III; (D) Stage IV.

**FIGURE A1:**
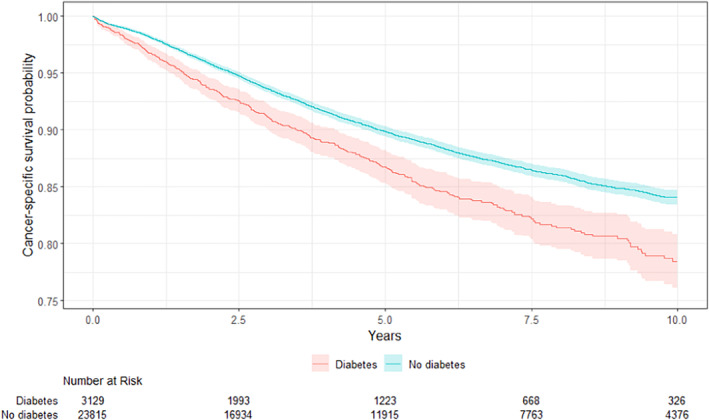
Breast cancer‐specific survival for women with diabetes, and women without diabetes (*p* value <.001).

For women with diabetes, the 5‐year and 10‐year KM estimates of breast cancer‐specific survival were 87% (95% CI: 85%–88%) and 79% (95% CI: 76%–81%) compared to 89% (95% CI: 89%–90%) and 84% (95% CI: 83%–85%) for women without diabetes (Table 2, Figure A1). When stratified by cancer stage, the breast cancer‐specific survival between these two groups was similar for women with stage I (5‐year and 10‐year: 98% and 95% for women with diabetes vs. 98% and 96% for women without diabetes), stage III (75% and 60% vs. 78% and 65%), and stage IV (26% and 11% vs. 25% and 13%) breast cancer (Figure 1). The exception was among those with stage II breast cancer, where women with diabetes had worse crude breast cancer‐specific survival outcomes than women without diabetes (90% and 79% vs. 92% and 85%).

**TABLE 2:** 5‐year and 10‐year breast cancer‐specific survival by cancer stage.

Subgroup	BC‐specific survival (%)	
	5‐year (95% CI)	10‐year (95% CI)
*All stages*		
Without diabetes	90 (89–90)	84 (83–85)
With diabetes	87 (85–88)	79 (76–81)
*Stage I*		
Without diabetes	98 (98–99)	96 (95–96)
With diabetes	98 (97–99)	95 (93–97)
*Stage II*		
Without diabetes	92 (91–92)	85 (84–86)
With diabetes	90 (89–92)	79 (75–84)
*Stage III*		
Without diabetes	78 (76–80)	65 (63–67)
With diabetes	75 (69–80)	60 (53–80)
*Stage IV*		
Without diabetes	25 (22–28)	13 (10–16)
With diabetes	26 (20–35)	11 (6–20)

### 3.3 Model results

The adjusted HR of breast cancer‐specific mortality for women with diabetes in comparison with women without diabetes was 0.99 (95% CI: 0.89–1.11), after adjustment for patient demographics, tumor characteristics, and cancer treatments (Table 3). Age at cancer diagnosis and cancer stage had the biggest impact on the survival difference between women with and without diabetes. When stratified by cancer stage, the breast cancer‐specific mortality between women with and without diabetes was similar across all stages (adjusted HR of 1.17, 1.07, 1.13, and 0.85 for stage I, II, III, and IV cancers). Among patients diagnosed with non‐metastatic breast cancer, the risk of metastatic relapse between women with and without diabetes were similar (adjusted HR of 0.96, 95% CI: 0.83–1.11). When stratified by stage, the respective adjusted HRs of metastatic relapse for women with diabetes in comparison with women without diabetes were 1.19, 0.96, and 0.90 for stage I, II, and III cancers, respectively (Table A2).

**TABLE 3:** Hazard ratio of breast cancer‐specific mortality and 95% CI for women with diabetes compared to women without diabetes.

Hazard ratio	Unadjusted	+ Age at diagnosis	+ Cancer stage	+ Other patient characteristics^a^	+ Treatments^b^
*All stages*	1.37 (1.23–1.53)	1.23 (1.10–1.37)	1.00 (0.90–1.12)	1.01 (0.90–1.13)	0.99 (0.89–1.11)
*By cancer stage*					
Stage I	1.16 (0.79–1.71)	1.04 (0.71–1.54)	‐	1.21 (0.81–1.79)	1.17 (0.79–1.74)
Stage II	1.31 (1.08–1.59)	1.11 (0.91–1.35)	‐	1.06 (0.86–1.30)	1.07 (0.87–1.31)
Stage III	1.17 (0.94–1.46)	1.06 (0.85–1.33)	‐	1.15 (0.91–1.45)	1.13 (0.90–1.43)
Stage IV	1.07 (0.89–1.28)	0.91 (0.76–1.10)	‐	0.90 (0.75–1.09)	0.85 (0.70–1.03)
*By ethnicity*					
Māori	1.09 (0.82–1.44)	1.12 (0.84–1.42)	0.98 (0.74–1.31)	1.07 (0.79–1.43)	1.01 (0.75–1.37)
Pacific	0.77 (0.58–1.01)	0.73 (0.55–0.97)	0.77 (0.58–1.03)	0.86 (0.64–1.16)	0.88 (0.65–1.19)
Asian	1.24 (0.83–1.86)	1.18 (0.77–1.80)	1.24 (0.82–1.89)	1.23 (0.80–1.91)	1.19 (0.76–1.86)
European/others	1.61 (1.40–1.85)	1.31 (1.14–1.51)	1.07 (0.93–1.23)	1.03 (0.90–1.20)	1.00 (0.87–1.16)

^a^ Year of diagnosis, menopausual status, ethnicity, mode of detection, subtype, cancer grade, and deprivation.

^b^ Primary surgery, radiotherapy, chemotherapy, endocrine therapy, and HER2 targeted therapy.

Among women with diabetes at the time of breast cancer diagnosis (Table 4), Asian women had better breast cancer‐specific survival compared to European/Other women, with an unadjusted HR of 0.50 (95% CI: 0.34–0.73) and an adjusted HR of 0.67 (95% CI: 0.45–1.01). After adjustment for patient demographics, tumor characteristics, and cancer treatments, among women with diabetes, the HR of breast cancer‐specific mortality was 1.12 (95% CI: 0.82–1.53) for Māori and 0.84 (95% CI: 0.61–1.15) for Pacific women when compared to European/Other women.

**TABLE 4:** Hazard ratio of breast cancer‐specific mortality and 95% CI compared to European/other, among patients with diabetes.

Hazard ratio	Unadjusted	+ Age at diagnosis	+ Cancer stage	+ Other patient characteristics^a^	+ Treatments^b^
European/others	Reference				
Māori	0.83 (0.62–1.10)	1.06 (0.79–1.43)	0.98 (0.73–1.32)	1.16 (0.85–1.58)	1.12 (0.82–1.53)
Pacific	0.87 (0.67–1.15)	1.14 (0.86–1.52)	0.76 (0.57–1.01)	0.87 (0.63–1.19)	0.84 (0.61–1.15)
Asian	0.50 (0.34–0.73)	0.61 (0.41–0.90)	0.70 (0.47–1.05)	0.71 (0.47–1.06)	0.67 (0.45–1.01)

^a^ Year of diagnosis, menopausual status, ethnicity, mode of detection, subtype, cancer grade, and deprivation.

^b^ Primary surgery, radiotherapy, chemotherapy, endocrine therapy, and HER2 targeted therapy.

## 4 DISCUSSION

Before consideration of confounding factors, women with diabetes had worse breast cancer‐specific survival than those without diabetes. Those differences were almost entirely explained by differences in age at diagnosis and cancer stage. We have recently observed that older women had a higher risk of having diabetes, and are associated with a higher risk of advanced stage: approximately 36% of women with diabetes at cancer diagnosis were aged 70 years or older compared to 21% of women without diabetes, while the proportion of stage II, III, and IV breast cancer among women with diabetes was all higher than women without diabetes (39.9%, 12.6% and 7.4% compared to 35.9%, 12.0%, and 5.0%, respectively).^11^ Most of the survival difference between women with and without diabetes can be attributed to the greater proportion of metastatic disease at diagnosis in women with diabetes which contributed to 38% of the breast cancer‐specific deaths found in this study.

A greater proportion of women with diabetes are more likely to die from other causes of death unrelated to breast cancer. Among women with diabetes, 56% of deaths were from other causes, and this was 40% in women without diabetes. The difference may be attributed to the comorbidities, age, and diabetes complications in women with diabetes. At breast cancer diagnosis, 82% of women with diabetes in our cohort had at least one comorbidity compared to 46% of women without diabetes (Table A1). This finding is consistent with another study looking at the global trends of macrovascular and microvascular complications.^26^ This study showed that CVD is the principal cause of death among diabetes patients, and adults with diabetes have 2–4 times increased CVD risk when compared with adults without diabetes.^26^ Diabetes is associated with a 75% increase in mortality rates among adults, with CVD as a significant contributor to the increased mortality in individuals with diabetes.^26^

Of those women with diabetes, we did not find any differences in survival between Māori/Pacific women and European/Other. This differs from what we had found in a previous study including all women with breast cancer,^6^ showing that Māori and Pacific women had a higher risk of excess mortality from breast cancer, of which 75% and 99% respectively were explained by demographic and clinical differences at diagnosis. The most important contributor was late stage at diagnosis in Māori and Pacific women.^6^ However, among women with diabetes, the proportion of women having metastatic disease at diagnosis did not differ substantially by ethnicity: 6.9% for Māori, 9.0% for Pacific, and 7.5% for Europeans. When diagnosed with stages I–III breast cancer, Māori or Pacific ethnicity did not influence the risk of developing recurrent metastatic disease,^27^ and therefore did not show disparities in cancer‐specific survival.

Our earlier studies found notable disparities in the treatment of breast cancer between women with and without diabetes. Specifically, diabetes is associated with a reduced likelihood of undergoing breast reconstruction following mastectomy. Additionally, it contributes to prolonged delays in surgery for breast cancer patients.^2^ Women with diabetes were also undertreated with chemotherapy,^28^ but had better adherence to endocrine therapy than women without diabetes.^13^, ^14^ Nevertheless, this study indicates that despite observed treatment disparities between women with diabetes and those without, these differences did not translate into discernible disparities in breast cancer survival after adjustments for age and cancer stage. This aligns with findings from a Canadian study, which reported that breast cancer patients with type II diabetes were 7% less likely to receive chemotherapy and 3% less likely to undergo radiation, but no significant difference in all‐cause mortality was identified after adjusting for comorbidities.^29^

The strength of this study is that the results were from a population‐based database, which provides a robust foundation for generating results. This richness of this database enhances the reliability and generalizability of the study findings, allowing for a more in‐depth exploration and analysis of the relationship between diabetes and breast cancer outcomes. The linkage to the VDR enabled us to identify patients with diabetes at breast cancer diagnosis, and the linkage to the mortality datasets provided detailed cause of death data to examine the survival difference in depth. Study limitations include a lack of data on body mass index (BMI), exercise, alcohol consumption, tobacco consumption, and diet that may provide reasonably strong residual confounding after conditioning on diabetes status. The comparison of breast cancer‐specific survival between the two groups may be affected by the competing risk of death, with women with diabetes being more likely to die of other causes (including comorbidities and diabetes complications) than breast cancer. Therefore, we had adjusted the effects of comorbidities on survival using the C3 index score excluding diabetes.

## 5 CONCLUSIONS

While crude differences in survival were identified for women with diabetes compared to women without diabetes, these were attributable to women with diabetes having an older age profile and more advanced disease at diagnosis. We did not find substantive differences in survival between the two groups due to differences in treatment once we adjusted for differences in age and stage.

## AUTHOR CONTRIBUTIONS

**Chunhuan Lao:** Conceptualization; methodology; data curation; writing – original draft; writing – review and editing; funding acquisition; software; formal analysis; validation; visualization. **Ross Lawrenson:** Conceptualization; methodology; supervision; writing – original draft; writing – review and editing; investigation; funding acquisition; formal analysis. **James Stanley:** Methodology; writing – review and editing; supervision; investigation; funding acquisition. **Andrea Teng:** Methodology; investigation; writing – review and editing; funding acquisition. **Marion Kuper‐Hommel:** Methodology; investigation; writing – review and editing. **Ian Campbell:** Methodology; investigation; writing – review and editing; supervision. **Jeremy Krebs:** Methodology; investigation; writing – review and editing. **Dianne Sika‐Paotonu:** Methodology; investigation; writing – review and editing. **Jonathan Koea:** Methodology; investigation; writing – review and editing. **Ineke Meredith:** Methodology; investigation; writing – review and editing. **Jason Gurney:** Funding acquisition; conceptualization; methodology; writing – review and editing; project administration; resources.

## CONFLICT OF INTEREST STATEMENT

The authors have stated explicitly that there are no conflicts of interest in connection with this article.

## ETHICS STATEMENT

Ethics approval for the study was granted through the University of Waikato Human Research Ethics Committee (reference: HREC(Health)2021#89).

## Data Availability

The data that support the findings of this study are available on request from the corresponding author. The data are not publicly available due to privacy or ethical restrictions.

## Appendix

**TABLE A1:** Patient demographics and tumor characteristics between women with diabetes, and without diabetes.

Subgroup	Without diabetes		With diabetes		Total	
*Year of diagnosis*						
2005–2009	4586	(19.2%)	453	(14.4%)	5039	(18.7%)
2010–2014	7799	(32.7%)	919	(29.3%)	8718	(32.3%)
2015–2020	11 446	(48.0%)	1765	(56.3%)	13 211	(49.0%)
*Ethnicity*						
Māori	2173	(9.1%)	547	(17.4%)	2720	(10.1%)
Pacific	1196	(5.0%)	533	(17.0%)	1729	(6.4%)
Asian	1899	(8.0%)	394	(12.6%)	2293	(8.5%)
European/Others	18 563	(77.9%)	1663	(53.0%)	20 226	(75.0%)
*Age (years)*						
<45	3291	(13.8%)	115	(3.7%)	3406	(12.6%)
45–59	9691	(40.7%)	866	(27.6%)	10 557	(39.1%)
60–69	5878	(24.7%)	988	(31.5%)	6866	(25.5%)
70–79	2948	(12.4%)	684	(21.8%)	3632	(13.5%)
80+	2023	(8.5%)	484	(15.4%)	2507	(9.3%)
*Menopausal status*						
Pre‐menopausal	7451	(31.3%)	382	(12.2%)	7833	(29.0%)
Peri‐menopausal	1210	(5.1%)	83	(2.6%)	1293	(4.8%)
Post‐menopausal	14 973	(62.8%)	2661	(84.8%)	17 634	(65.4%)
Unknown	197	(0.8%)	11	(0.4%)	208	(0.8%)
*Deprivation quintile*						
1 (least deprived)	5490	(23.0%)	412	(13.1%)	5902	(21.9%)
2	5291	(22.2%)	505	(16.1%)	5796	(21.5%)
3	4943	(20.7%)	593	(18.9%)	5536	(20.5%)
4	4547	(19.1%)	705	(22.5%)	5252	(19.5%)
5 (most deprived)	3384	(14.2%)	916	(29.2%)	4300	(15.9%)
Unknown	176	(0.7%)	6	(0.2%)	182	(0.7%)
*Cancer stage*						
Stage I	11 241	(47.2%)	1259	(40.1%)	12 500	(46.4%)
Stage II	8554	(35.9%)	1253	(39.9%)	9807	(36.4%)
Stage III	2856	(12.0%)	394	(12.6%)	3250	(12.1%)
Stage IV	1180	(5.0%)	231	(7.4%)	1411	(5.2%)
*Cancer grade*						
1	4862	(20.4%)	520	(16.6%)	5382	(20.0%)
2	10 343	(43.4%)	1305	(41.6%)	11 648	(43.2%)
3	6571	(27.6%)	808	(25.8%)	7379	(27.4%)
Unknown	2055	(8.6%)	504	(16.1%)	2559	(9.5%)
*Subtype*						
Luminal A	14 410	(60.5%)	1980	(63.1%)	16 390	(60.8%)
Luminal B HER2−	2376	(10.0%)	287	(9.1%)	2663	(9.9%)
Luminal B HER2+	2541	(10.7%)	273	(8.7%)	2814	(10.4%)
HER2+ non‐Luminal	1047	(4.4%)	114	(3.6%)	1161	(4.3%)
Triple negative	2007	(8.4%)	235	(7.5%)	2242	(8.3%)
Unknown	1450	(6.1%)	248	(7.9%)	1698	(6.3%)
*Mode of detection*						
Screen‐detected	9926	(41.7%)	1264	(40.3%)	11 190	(41.5%)
Symptomatic	13 412	(56.3%)	1795	(57.2%)	15 207	(56.4%)
Unknown	493	(2.1%)	78	(2.5%)	571	(2.1%)
Total	23 831		3137		26 968	

**TABLE A2:** Hazard ratio of metastatic relapse for patients with diabetes compared to patients without diabetes.

Hazard ratio	Unadjusted	+ Age at diagnosis	+ Cancer stage	+ Other patient characteristics^a^	+ Treatments^b^
*All stages*	0.99 (0.86–1.13)	1.06 (0.92–1.22)	0.96 (0.83–1.10)	0.97 (0.84–1.13)	0.96 (0.83–1.11)
*By cancer stage*					
Stage I	1.01 (0.70–1.46)	1.06 (0.73–1.53)	‐	1.22 (0.83–1.78)	1.19 (0.82–1.74)
Stage II	0.99 (0.81–1.20)	1.01 (0.82–1.23)	‐	0.98 (0.79–1.21)	0.96 (0.78–1.19)
Stage III	0.81 (0.64–1.01)	0.87 (0.69–1.10)	‐	0.89 (0.70–1.13)	0.90 (0.70–1.14)

^a^ Year of diagnosis, menopausual status, ethnicity, mode of detection, subtype, cancer grade, and deprivation.

^b^ Primary surgery, radiotherapy, chemotherapy, endocrine therapy, and HER2 targeted therapy.
